# Comparative efficacy of repurposed drugs lopinavir-ritonavir and darunavir-ritonavir in hospitalised COVID-19 patients: insights from a tertiary centre cohort

**DOI:** 10.3389/fcimb.2024.1496176

**Published:** 2025-01-16

**Authors:** Dóra Paróczai, András Bikov, Andreea Blidaru, Emanuel Bobu, Ana Lascu, Cristian Ion Mot, Stefan Mihaicuta, Stefan Frent

**Affiliations:** ^1^ Department of Medical Microbiology, University of Szeged, Szeged, Hungary; ^2^ Albert Szent-Györgyi Health Center, Pulmonology Clinic, University of Szeged, Deszk, Hungary; ^3^ North West Lung Centre, Wythenshawe Hospital, Manchester University NHS Foundation Trust, Manchester, United Kingdom; ^4^ Division of Immunology, Immunity to Infection and Respiratory Medicine, University of Manchester, Manchester, United Kingdom; ^5^ Department of Infectious Diseases, Infectious Diseases and Pulmonology Clinical Hospital, Timisoara, Romania; ^6^ Department of Pulmonology, University of Medicine and Pharmacy Timisoara, Timisoara, Romania; ^7^ Department of Functional Sciences, Discipline of Pathophysiology, Centre for Translational Research and Systems Medicine, University of Medicine and Pharmacy Timisoara, Timisoara, Romania; ^8^ Institute for Cardiovascular Diseases of Timisoara, Clinic for Cardiovascular Surgery, Timisoara, Romania; ^9^ ENT Department, Municipal Emergency Hospital Timisoara, Timisoara, Romania; ^10^ Department of Surgery, University of Medicine and Pharmacy Timisoara, Timisoara, Romania; ^11^ Centre for Research and Innovation in Precision Medicine of Respiratory Diseases, Department of Pulmonology, University of Medicine and Pharmacy Timisoara, Timisoara, Romania

**Keywords:** COVID-19, darunavir, lopinavir, propensity score matching, ritonavir

## Abstract

**Background:**

Drug repurposing has become a widely adopted strategy to minimise research time, costs, and associated risks. Combinations of protease inhibitors such as lopinavir and darunavir with ritonavir have been repurposed as treatments for COVID-19. Although lopinavir-ritonavir (LPV/r) and darunavir-ritonavir (DRV/r) have shown *in vitro* efficacy against COVID-19, the results in human studies have been inconsistent. Therefore, our objective was to compare the efficacy of LPV/r and DRV/r in COVID-19 patients admitted to a tertiary centre in Romania.

**Research design and methods:**

A clinical dataset from 417 hospitalised patients was analysed. Patients were assigned to the LPV/r, DRV/r, or control (standard-of-care) group based on clinical decisions made by the attending infectious disease specialists, aligned with national treatment protocols. Kaplan-Meier and Cox proportional hazards regression analyses were conducted to compare in-hospital mortality and to identify factors associated with clinical improvement or fatal outcomes.

**Results:**

By day 10, more patients showed improvement with LPV/r and DRV/r (p=0.03 and 0.01, respectively), but only LPV/r was associated with improved survival compared to the control group (p=0.05). Factors associated with mortality included male gender (HR: 3.63, p=0.02), diabetes (HR: 2.49, p=0.03), oxygen saturation below 90% at admission (HR: 5.23, p<0.01), high blood glucose levels (HR: 3.68, p=0.01), age (HR: 1.04, p=0.02), and more than 25% lesion extension on chest CT scan (HR: 2.28, p=0.03).

**Conclusions:**

LPV/r, but not DRV/r, showed a survival benefit in patients hospitalised with COVID-19, but these findings deserve further investigation in a randomised clinical trial.

## Introduction

The COVID-19 pandemic, caused by severe acute respiratory syndrome coronavirus 2 (SARS-CoV-2), emerged in late 2019 (Kumar et al., 2021) and evolved into a global health crisis within the first few months of 2020 (Mallah et al., 2021). Despite extensive research, the effectiveness of some proposed treatments for COVID-19 remains uncertain. The pandemic affected over 650 million individuals worldwide, resulting in more than 6 million deaths. The burden on healthcare systems, as well as the lack of efficient medications, prompted drug regulators, including agencies such as The Food and Drug Administration (FDA), to authorise the use of repurposed drugs and off-label medications (Singh et al., 2020). Humans across the globe are regularly infected with endemic coronaviruses, which typically result in respiratory illnesses with mild symptoms (Li et al., 2005). These viruses have not been deemed a significant public health threat, and thus the development of specific antiviral treatments or preventive vaccines was not prioritised. Consequently, when SARS-CoV-2 appeared, no specific antiviral treatments for coronavirus diseases, including COVID-19, were available. Traditional methods for discovering new antiviral compounds and developing new therapeutic options are lengthy and complex, often taking several years. In this scenario, drug repurposing has emerged as a promising and potentially valuable strategy for identifying already approved drugs for the treatment of other diseases, including COVID-19.

Drug repurposing offers a cost-effective and time-efficient alternative to developing new drugs. As a result, medications like remdesivir, favipiravir, umifenovir, lopinavir, ritonavir, and darunavir were utilised in clinical practice to treat COVID-19. Some of these drugs such as lopinavir, remdesivir and darunavir derivates demonstrated inhibitory effects on SARS-CoV-2 replication *in vitro* on cell cultures (Choy et al., 2020; Wang et al., 2020; Ma et al., 2022). Protease inhibitors, originally developed to target aspartate protease in HIV treatment, have been among the most extensively studied repurposed drugs and were found to inhibit the 3C-like protease of SARS-CoV-2 (Nutho et al., 2020).

Both LPV/r and DRV/r target the viral protease necessary for SARS-CoV-2 replication. However, LPV/r primarily inhibits the 3CL-like protease, while DRV/r demonstrates activity at higher concentrations and was initially designed to target only the HIV-1 protease. An *in vitro* study clarified that darunavir derivates can also inhibit 3CL-like protease like LPV/r (Ma et al., 2022). However, they have distinct pharmacodynamic properties: their inhibitory action differs in affinity and efficacy for SARS-CoV-2 protease target, with LPV/r showing greater efficacy at clinically achievable concentrations (Choy et al., 2020; Kang et al., 2020).

Moreover, protease inhibitors showed *in vitro* efficacy against coronaviruses causing severe acute respiratory syndrome (SARS) and Middle-East respiratory syndrome (MERS) (Chu et al., 2004). Studies conducted to evaluate the protease inhibitors combination of lopinavir-ritonavir with ribavirin reduced the mortality and viral load of SARS patients (Chu et al., 2004). Similarly, in MERS, lopinavir-ritonavir was effective both *in vitro* and in animal models, decreasing the viral load and improving clinical and radiological outcomes (de Wilde et al., 2014; Chan et al., 2015). Although the clinical impact of lopinavir-ritonavir in SARS or MERS was scarce, *in vitro* and animal studies revealed the potential inhibitory effect on SARS-CoV-2. Based on the structural similarities of the coronaviruses, lopinavir and ritonavir showed favourable inhibitory action on the replication of SARS-CoV-2 *in vitro* (Choy et al., 2020; Kang et al., 2020). Another protease inhibitor, darunavir-cobicistat was also found to have *in vitro* inhibitory effect on SARS-CoV-2, but only at higher concentrations (Yamamoto et al., 2020). The initiation of lopinavir-ritonavir and darunavir-cobicistat into COVID-19 treatments was based on the previous experience in SARS and MERS, however, in the last two years several clinical trials were conducted in SARS-CoV-2 infection and most showed no significant improvement in mortality, viral load, hospital stay or the need for ventilation support. Of note, the design of some of these clinical trials raised several bias concerns in terms of patient enrolment and sample size, the start of anti-viral therapy after disease onset, underpowered statistics and the lack of control group (Cao et al., 2020b; Patel et al., 2021). Lopinavir-ritonavir was abandoned as a therapeutic option based on these studies with few concerns, including the trial conducted by Cao et al., which failed to demonstrate a significant benefit of lopinavir-ritonavir in decreasing mortality rates (Cao et al., 2020a; Owa and Owa, 2020). However, patients receiving lopinavir-ritonavir showed lower 28-day mortality rates and had a shorter intensive care unit (ICU) stay with a median of 6 days, compared to 11 days in the standard of care group. Data of this trial were reanalysed and a 73% a posteriori probability of clinical improvement in case of the protease inhibitors was found (Carmona-Bayonas et al., 2020). Along with this observation, a report by Lim et al. confirmed the beneficial clinical effects of lopinavir-ritonavir (Lim et al., 2020).

To our knowledge, data is lacking on the efficacy of lopinavir-ritonavir compared to the standard of care and to an additional protease inhibitor alternative, darunavir-ritonavir combination. Thus, we conducted a retrospective study to assess the differences between various protease inhibitors on comparable patient groups, from a Romanian cohort of patients hospitalised for COVID-19.

## Patients and methods

### Study design

We conducted a retrospective, single-site, tertiary care center-based, observational study to compare several clinical outcomes in patients taking lopinavir-ritonavir (Kaletra, 200 mg/50 mg, 1 tablet orally twice daily) or darunavir/ritonavir (800 mg darunavir/100 mg ritonavir 1 tablet once daily). We included consecutive patients hospitalised for COVID-19 in the Pulmonology and Infectious Diseases departments of our hospital, between July and October 2020. Concomitantly hospitalised COVID-19 patients, who received standard of care only including antibiotics, anticoagulants, dexamethasone and oxygen therapy via nasal cannula or oxygen mask, served as our controls. Patients were assigned to receive lopinavir/ritonavir, darunavir/ritonavir or standard of care only based on the infectious diseases’ specialist advice concordant with a national treatment protocol, and the treatment duration with the protease inhibitors was 7 - 14 days. Of note, the antiviral treatment in eligible patients was started on or the next day of hospital admission. The research project was approved by the hospital’s Ethics Committee on 16^th^ of November 2020, on the condition of respect for the confidentiality of personal data and compliance with all applicable data protection laws and regulations. Given the retrospective design of data collection, there was no request from the Ethics Committee to obtain informed consent from the subjects included in the study. The research was conducted in accordance with the principles of the Declaration of Helsinki and its later amendments.

### Study population and data collection

We collected data from a cohort compromising 824 hospitalised COVID-19 patients, with confirmed SARS-CoV-2 infection. Male and non-pregnant female patients older than 18 years were eligible if they needed hospitalisation, had a positive quantitative real-time polymerase chain reaction (RT-PCR) for SARS-CoV-2 infection, suggestive symptomatology and chest imaging. Exclusion criteria included pregnancy, prior use of protease inhibitors, necessity of invasive ventilation before considering antivirals, history of known cardiac arrhythmias, known drug allergy, a history of severe liver conditions such as cirrhosis and/or elevated alanine aminotransferase or aspartate aminotransferase level, and inability to swallow the medications.

The diagnosis of COVID-19 was confirmed by the detection of SARS-CoV-2 nucleocapsid genes with Exicycler™ 96 – Bioneer (Korea) RT-PCR system. We collected medical data, including demographics, laboratory tests results, chest CT scan reports, symptomatology, current medication use and comorbidities. Data were obtained from the hospital available documents (i.e., patients’ files, discharge letters, electronic database) as recorded by the treating physicians and were assessed for accuracy by the investigators. As a standard of care, all COVID-19 hospitalised patients underwent a chest CT scan upon admission, which was interpreted by experienced radiologists. The extension of lung lesions was graded as mild (<25%), moderate (25-50%) or severe (>50%).

### Statistical analysis

Propensity score matching based on age, gender, oxygen use, steroid therapy and chest computed tomography (CT) imaging was used to divide the source population consisting of 824 patients. As 139 patients received LPV/r combination, all three groups (LPV/r, DRV/r, and control) comprised in the end the same number of patients.

Baseline characteristics were expressed as average ± standard deviation (SD) or proportions. The time to death or clinical improvement were assessed by Kaplan-Meier plot using log-rank test. In order to assess the relationship between mortality and drug administration, we used the Cox proportional hazard model and hazard ratios were shown with 95% CIs. All p<0.05 values were considered significant, adjusted p values were calculated with the Bonferroni method. We performed sensitivity analyses in pre-defined subgroups [male gender, age>50 years, >25% lesions on chest CT, presence of obesity, arterial hypertension or diabetes, high blood glucose level, low SaO2, high C- reactive protein (CRP) level, and the use of oxygen, dexamethasone and low molecular weight heparin (LMWH)]. Statistics were performed with R version 4.2.2 (R Project for Statistical Computing) and the Graphpad Prism 8.0.1 software.

## Results

### Patient characteristics

**Figure 1:**
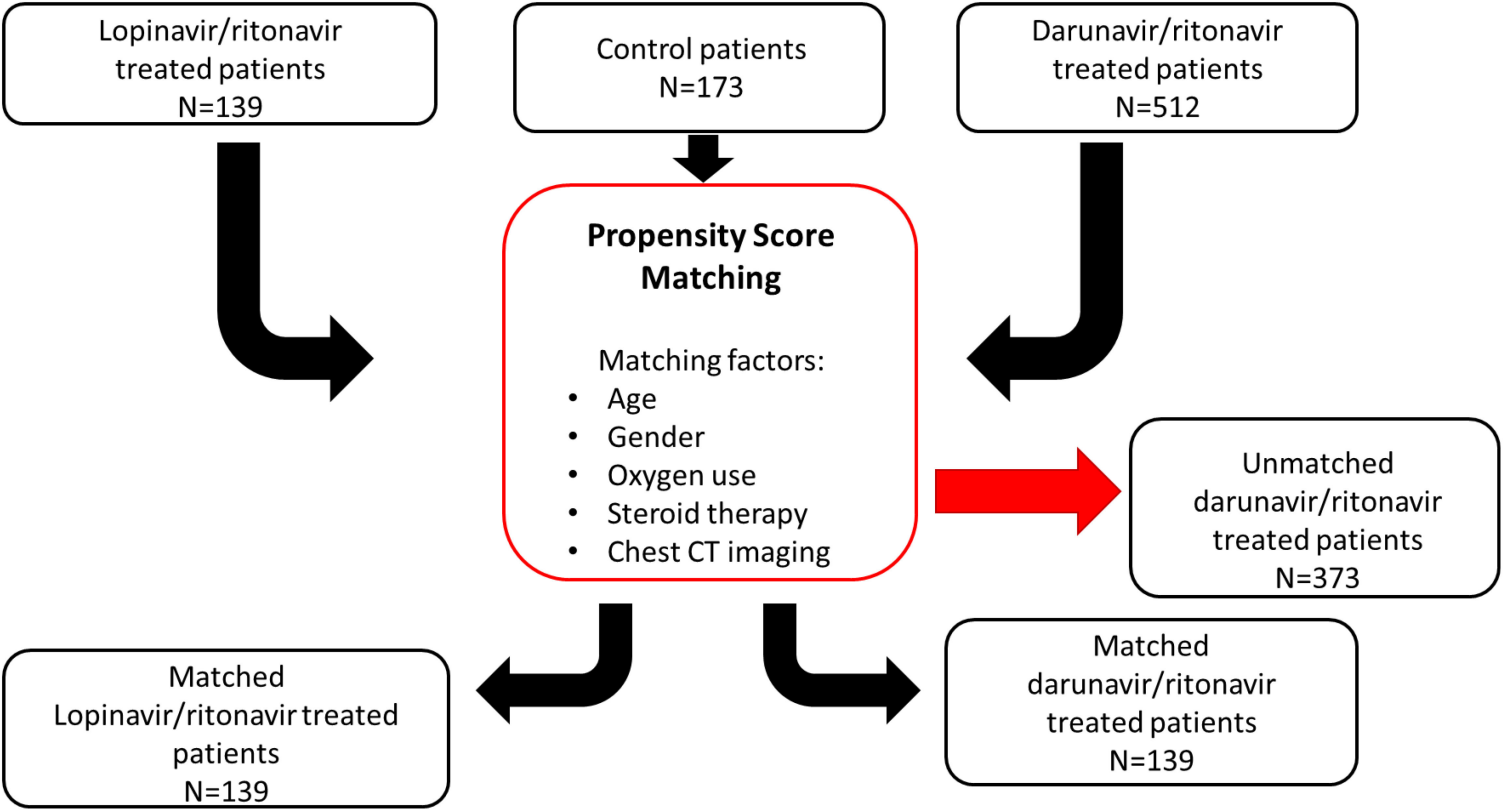
Flowchart for the patient selection pathway with propensity score matching method. A population of 824 patients was assigned using propensity score matching based on age, sex, oxygen use, steroid therapy, and chest CT imaging. 139 out of 824 patients received LPV/r combination, so the three groups (LPV/r, DRV/r, and control) comprised in the end the same number of patients.

The flowchart for patient selection is summarised on **Figure 1**.

With respect to age, gender, and comorbidities, the three groups were generally balanced. **Table 1** summarised the pre-treatment demographic and clinical characteristics. Patients in the LPV/r showed less 25-50% lesion extension on chest CT, compared to DRV/r group (p<0.05) and fewer patients had obesity or diabetes, compared to the control and DRV/r group. In both treatment arms gastrointestinal symptoms, headache and myalgia were the most common symptoms and occurred more frequently than in the control group (**Table 1**).

**Table 1:** General characteristics of COVID-19 patients, assigned to lopinavir/ritonavir (LPV/r), darunavir/ritonavir (DRV/r) use or standard care.

	Control N=139	LPV/r N=139	Unadjusted P value	Adjusted P value	DRV/r N=139	Unadjusted P value	Adjusted P value
Age (mean, SD)	50.5 ± 16.9	50.2 ± 15.3	0.98	ns	52.5 ± 5.8	0.45	ns
Male sex No (%)	75 (54%)	62 (45%)	0.06	ns	72 (52%)	0.36	ns
Smoking history	14 (10.1%)	21 (15.8%)	0.1	ns	31 (22.3%)	0.44	ns
Disease severity on chest CT No (%)							
*Grade <25%*	101 (73%)	95 (68%)	0.18	ns	69 (49%)	<0.01	
*Grade 25-50%*	29 (21%)	37 (26%)	0.16		62 (45%)	<0.01	
*Grade >50%*	9 (6%)	7 (5%)	0.35		8 (5.7%)	0.45	
Hospital-onset COVID-19	4 (2.9%)	8 (5.8%)	0.11	ns	2 (1.5%)	0.2	ns
Coexisting conditions No (%)							
*Diabetes*	30 (21.6%)	15 (10.8%)	0.007	ns	31 (22.3%)	0.44	ns
*Arterial hypertension*	50 (35.6%)	52 (37.4%)	0.4	ns	62 (44.6%)	0.07	ns
*Chronic kidney disease*	5 (3.6%)	1 (0.7%)	0.05	ns	6 (4.3%)	0.38	ns
*Obesity*	35 (25.2%)	18 (13%)	0.004	0.116	67 (48.2%)	<0.001	0.01
*Asthma*	4 (2.8%)	8 (5.7%)	0.12	0.99	13 (9.5%)	0.01	0.39
*Other cardiovascular comorbidities*	20 (14.4%)	16 (11.5%)	0.23	ns	21 (15.1%)	0.43	ns
*Other pulmonary conditions*	6 (4.3%)	8 (5.7%)	0.29	Ns	11 (7.9%)	0.1	ns
*Neurological disorders*	3 (2.1%)	8 (5.7%)	0.06	Ns	10 (7.2%)	0.02	0.78
Symptoms							
*Asymptomatic*	18 (13%)	4 (2.8%)	0.001	0.029	6 (4.31%)	0.005	0.19
*Asthenia*	47 (33.8%)	53 (38.1%)	0.22	Ns	54 (38.8%)	0.19	ns
*Fever*	55 (39.5%)	67 (48.2%)	0.07	Ns	84 (60.4%)	<0.001	0.01
*Sweats*	10 (7.2%)	24 (17.3%)	0.005	0.145	15 (10.8%)	0.14	ns
*Fatigue*	69 (49.6%)	73 (52.5%)	0.31	Ns	63 (45.3%)	0.23	ns
*Dyspnea*	35 (25.1%)	27 (19.4%)	0.12	Ns	55 (39.5%)	0.005	0.19
*Dry cough*	66 (47.5%)	77 (55.4%)	0.09	Ns	86 (61.8%)	0.008	0.31
*Productive cough*	9 (6.5%)	16 (11.5%)	0.07	Ns	17 (12.2%)	0.05	ns
*Chills*	17 (12.2%)	31 (22.3%)	0.01	0.3	43 (30.9%)	<0.001	0.003
*Headache*	33 (23.7%)	57 (41%)	0.001	0.03	50 (35.9%)	0.01	0.39
*Diarrhoea*	10 (7.2%)	16 (11.5%)	0.1	Ns	24 (17.3%)	0.005	ns
*Nausea*	7 (5%)	19 (13.7%)	0.007	0.2	22 (15.8%)	0.001	0.04
*Vomiting*	7 (5%)	12 (8.6%)	0.11	Ns	18 (12.9%)	0.01	0.4
*Altered general condition*	73 (52.5%)	73 (52.5%)	0.5	Ns	108 (77.7%)	<0.001	0.003
*Loss of appetite*	15 (10.8%)	29 (20.1%)	0.01	0.29	9 (6.5%)	0.1	ns
*Ageusia*	15 (10.8%)	13 (9.3%)	0.34	Ns	24 (17.3%)	0.06	ns
*Dysphagia*	19 (13.7%)	27 (19.4%)	0.09	Ns	26 (18.7%)	0.12	ns
*Myalgia*	23 (16.5%)	35 (25.2%)	0.03	0.87	56 (40.3%)	<0.001	0.004

Data are expressed as number (percentage).

**Table 2** shows the main clinical characteristics at admission and discharge including vital parameters, laboratory tests results and adjuvant therapies. Heart rate, blood pressure, body temperature and SaO2 did not show significant differences between the groups both at admission and at discharge. Although more patients had known diabetes in the DRV/r group, the LPV/r and control group had comparable glycaemia at admission. In both antiviral groups, dexamethasone and LMWH were more frequently administered, and patients receiving DRV/r more commonly needed oxygen supplementation (**Table 2**).

**Table 2:** Patient status and treatments administered at the time of enrolment and discharge.

	Control	LPV/r	p (vs. control)	DRV/r	p (vs. control)
SaO2 (admission)	95.2 ± 4.4	95 ± 5.7	0.86	94.6 ± 3.4	0.99
Low SaO2 at admission (<90%)	12 (8.6%)	13 (9.4%)	0.4	20 (14%)	0.07
SaO2 (discharge)	95.4 ± 6.2	95.7 ± 5.7	0.92	94.8 ± 6.3	0.81
Blood pressure admission (systole, mmHg)	129.31 ± 18.79	128.48 ± 17.52	0.92	130.82 ± 19.54	0.77
Blood pressure admission (diastole, mmHg)	80.34 ± 12.35	80.52 ± 10.23	0.99	82.76 ± 12.47	0.2
Heart rate admission	84.86 ± 14.13	85.06 ± 14.55	0.99	87.46 ± 15.54	0.31
Body temperature admission (°C)	36.21 ± 2.85	36.57 ± 0.71	0.21	36.51 ± 0.65	0.33
Blood pressure discharge (systole, mmHg)	124.63 ± 15.63	125.43 ± 16.13	0.91	126.39 ± 18.79	0.66
Blood pressure discharge(diastole, mmHg)	78.58 ± 11.0	78.51 ± 10.56	0.99	80.01 ± 12.22	0.54
Heart rate discharge	79.52 ± 12.32	81.32 ± 13.78	0.51	80.67 ± 14.60	0.76
Body temperature discharge (°C)	36.32 ± 0.46	36.27 ± 0.46	0.97	36.31 ± 0.36	0.99
Na (mmol/l)	135.88 ± 3.95	136.98 ± 3.07	0.047	136.68 ± 4.16	0.14
K (mmol/l)	4.26 ± 0.63	4.12 ± 0.51	0.12	4.34 ± 0.65	0.71
CRP (mg/ml)	39.3 ± 59.2	37.5 ± 58.5	0.8	63.7 ± 88.6	<0.01
Glycaemia (admission)	136.9 ± 79.8	136.7 ± 73.6	0.99	145.9 ± 73	0.55
Impaired glucose >110 mg/dl	54 (39%)	67 (48%)	0.065	87 (62.6%)	<0.01
High glucose >125 mg/dl	41 (29.5%)	48 (34.5%)	0.18	87 (62.6%)	<0.01
Fibrinogen	4.43 ± 1.48	4.52 ± 1.78	0.96	5.07 ± 4.2	0.14
GOT (U/ml)	27.98 ± 19.89	40.50 ± 71.31	0.76	59.61 ± 238.26	0.52
GPT (U/ml)	35.88 ± 36.65	68.84 ± 192.92	0.07	54.74 ± 66.17	0.41
Oxygen therapy	33 (23.7%)	30 (21.6%)	0.33	74 (53.2%)	<0.01
Ceftriaxone	34 (24.5)	24 (17.3%)	0.06	55 (39.6%)	<0.01
Azithromycin	56 (40.3%)	58 (41.7%)	0.4	68 (48.9%)	0.07
Dexamethasone	58 (41.7%)	59 (42.4%)	0.45	116 (83.4%)	<0.01
LMWH	84 (60.4%)	110 (79.1%)	<0.01	136 (97.8%)	<0.01

Data are expressed as number (percentage) or mean± standard deviation (SD). CRP, C-reactive protein; GOT, glutamate oxaloacetate transaminase; GPT, glutamate pyruvate transaminase; LMWH, Low molecular weight heparin; SaO2, arterial oxygen saturation.

### Outcomes

Although the mean duration of hospitalisation and the time to clinical improvement were significantly higher in both treated groups compared to controls (p<0.01), in-hospital mortality rate was decreased by lopinavir/ritonavir compared to the control and darunavir/ritonavir groups. As compared to the control group, univariable hazard ratios for death were 0.39 (95% CI 0.19-0.99) and 1.49 (95% CI 0.71-3.12) for LPV/r and for DRV/r, respectively (**Table 3**).

**Table 3:** Mortality data of study population by treatment allocation.

	Control N=139	LPV/r N=139	Difference	Adj.p (vs. control)	DRV/r N=139	Difference	Adj.p (vs. control)	LPV/r vs. DRV/r difference	Adj.p LPV/r vs. DRV/r
Days spent in hospital	8.09 ± 6.0	10.63 ± 5.5	-2.54 (-4.24 to -0.83)	<0.001	12.83 ± 6.4	-4.73(-6.42 to -3.044)	<0.001	-2.193 (-3.89 to -0.48)	0.007
Time to clinical improvement (average days ± SD)	7.9 ± 5.9	10.6 ± 5.4	-2.69 (-4.41 to -0.97)	<0.001	11 ± 6.3	-3.13 (-4.87 to -1.37)	<0.001	-0.43 (-2.16 to 1.30)	0.82
In-hospital mortality	10 (7.2%)	5 (3.6%)		0.09	14 (10%)		0.19		0.016
Time to death	10.1 ± 6.7	13.8 ± 8.4	-3.7 (-13.68 to 6.27	0.63	13.4 ± 7.1	-3.32 (-10.87 to 4.21)	0.52	0.37 (-9.11 to 9.8)	0.99
Univariate analysis (HR, 95% CI)	–	0.39 (0.19-0.99)	–	0.04	1.49 (0.71-3.12)	–	0.28		

Data is expressed as mean ± SD. CI, confidence interval; HR, hazard ratio.

Several confounding factors may have contributed due to the retrospective nature of the study, thus the Cox proportional hazards model was adjusted for significant and clinically relevant baseline variables. These factors include comorbidities, oxygen saturation, high glucose levels and radiological abnormalities. In this multivariate analysis, the association with decreased mortality rates in LPV/r group remained significant (HR=0.25, 95% CI 0.09-0.66), while the administration of DRV/r was associated with higher risk for mortality (HR=2.60, 95% CI 1.37-4.92) (**Table 4**).

**Table 4:** Hazard ratios for the risk factor of death in the study population.

	HR	CI 95%	p value
Age	1.04	1.00-1.07	0.02
Male sex	3.63	1.23-10.68	0.02
LPV/r	0.25	0.09-0.66	<0.01
DRV/r	2.60	1.37-4.92	<0.01
Obesity	1.49	0.65-3.43	0.34
Diabetes	2.49	1.05-5.58	0.03
Low SaO2 (<90%)	5.23	2.45-11.17	<0.001
High glucose >125 mg/dl	3.68	1.23-10.91	0.01
Grade1 lesion (<25%)	0.12	0.02-0.53	0.005
Grade3 lesion (>50%)	2.67	1.12-6.39	0.03
Dexamethasone	1.69	0.55-5.18	0.36
Oxygen therapy	1.37	0.57-3.31	0.47

Cox proportional hazard model was used to determine the relationship of clinical factors and in-hospital mortality in the treated and control groups (n=417). Hazard ratios are shown with 95% CIs. All COVID-19 hospitalised patients underwent a chest CT scan and the extension of lung lesions was graded as mild (<25%, Grade 1), moderate (25-50%, Grade 2) or severe (>50%, Grade 3).

**Figure 2:**
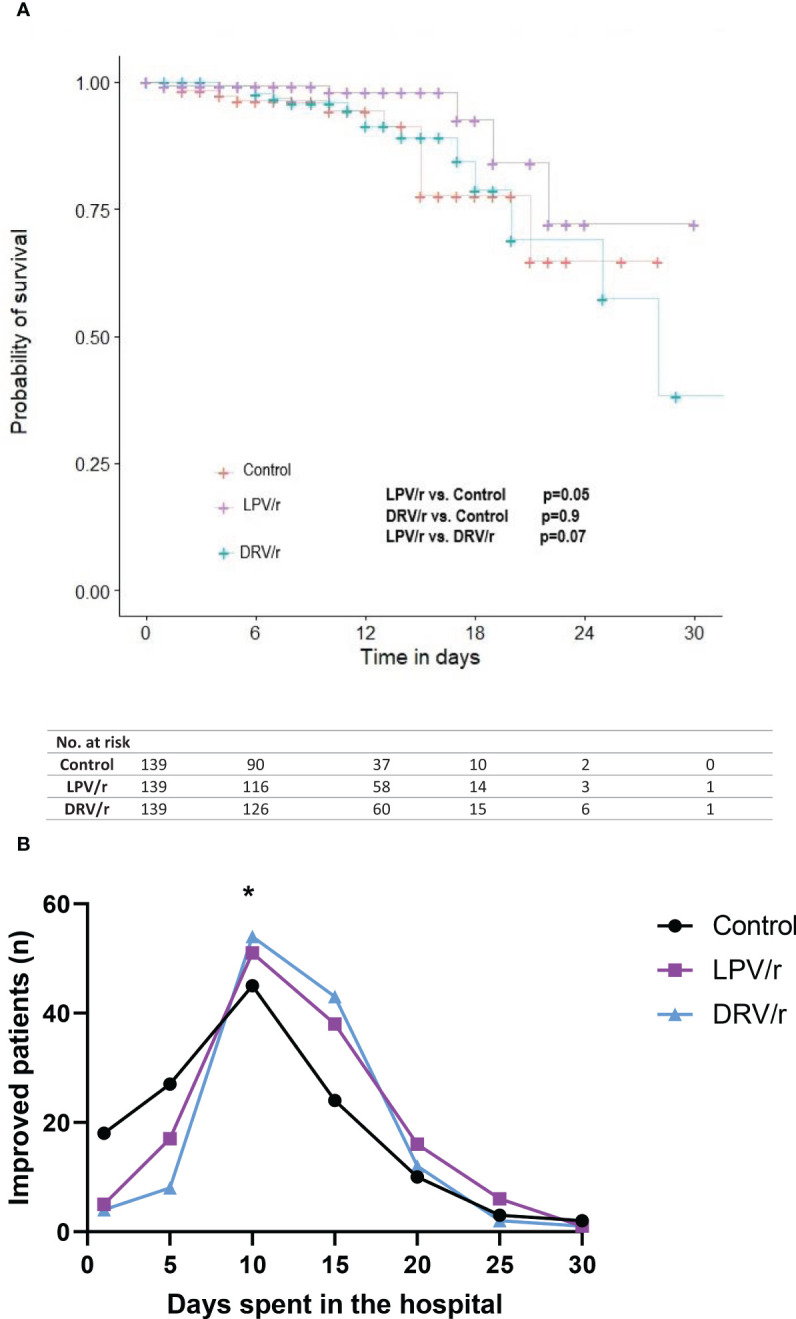
**(A)** Survival curves according to LPV/r or DRV/r use. A Kaplan-Meier analysis was performed to evaluate in-hospital mortality. Significance was calculated by using log-rank test. **(B)** Patient improvement on LPV/r or DRV/r therapy. Patient improvement was expressed according to the days patients spent in hospital. At day 10 both the LPV/r and DRV/r treated patients showed significant improvement compared to control participants. n= number of patients, *p<0.05.

Kaplan-Meier analysis on antiviral treatment which was used to compare survival curves using the log-rank test, revealed that unlike DRV/r, LPV/r was associated with higher probability of survival (**Figure 2A**). Interestingly, clinically improved alive patients showed significant improvement at day 10 with both LPV/r and DRV/r, but not with standard care (p< 0.05, **Figure 2B**). Clinically improved patients were defined as those who were alive and demonstrated stabilised vital signs, improved oxygen saturation, better laboratory markers, and readiness for discharge.

In a subgroup analysis, LPV/r demonstrated improved survival in the group of patients with >25% lesion extension on the chest CT scan, however, the presence of comorbidities such as arterial hypertension, diabetes and the need for oxygen therapy significantly decreased the benefits of LPV/r. (**Table 5**).

**Table 5:** Hazard ratios for mortality according to LPV/r and DRV/r use in different subgroups.

	HR (95% CI)	
Subgroups	LPV/r vs. control	DRV/r vs. control
Demographics		
Men (N=209)	0.45 (0.15-1.35)	0.71 (0.50-1.01)
Age >50 years (N=223)	0.54 (0.14-1.66)	0.78 (0.28-2.09)
Comorbidities		
Obesity		
Yes (N=120)	0.55 (0.06-5.13)	0.89 (0.28-2.74)
No (N=297)	0.35 (0.09-1.35)	0.93 (0.28-3.07)
Diabetes		
Yes (N=76)	0.93 (0.16-5.16)	2.10 (0.61-7.21)
No (N=341)	0.23 (0.06-0.96) p=0.044	0.61 (0.20-1.84)
Arterial hypertension		
Yes (N=164)	1.02 (0.26-3.89)	1.30 (0.42-4.05)
No (N=253)	0.07 (0.0086-0.67) P= 0.021	0.56 (0.17-1.88)
Patient status		
Degree of COVID-19 severity on chest CT scan		
Grade<25% (N=265)	NA (1)	NA (2)
Grade>25% (N=152)	0.35 (0.12-1.03) p=0.05	0.63 (0.27-1.48)
Impaired glucose metabolism (>110 mg/dl)		
Yes (N=208)	0.42 (0.12-1.51)	1.28 (0.48-3.39)
No (N=263)	0.18 (0.02-1.6)	NA (1)
Low SaO2 (<90%)		
Yes (N=45)	0.47 (0.11-1.97)	0.58 (0.16-2.02)
No (N=372)	0.29 (0.05-1.54)	1.49 (0.45-4.91)
High CRP (10 mg/dl)		
Yes (N=243)	0.29 (0.07-1.14) P=0.07	1.15 (0.44-2.80)
No (N=174)	NA	NA
Therapies		
Use of oxygen		
Yes (N=137)	0.65 (0.16-2.59)	1.14 (0.40-3.03)
No (N=280)	0.18 (0.03-1.12) p=0.05	0.62 (0.13-2.85)
Use of dexamethasone		
Yes (N=228)	0.73 (0.22-2.39)	1.16 (0.44-3.04)
No (N=189)	NA	NA
Use of LMWH		
Yes (N=330)	0.61 (0.18-2.00)	1.41 (0.54-3.69)
No (N=87)	NA	NA

A subgroup analysis was conducted to reveal the main risk factors in mortality according to protease inhibitor use. Hazard ratios are shown with 95% CIs. Values showed NA (not applicable) meaning the inability to calculate values due to low number of patients. CRP, C-reactive protein; LMWH, Low molecular weight heparin; SaO2, arterial oxygen saturation.

## Discussion

In this single centre, retrospective study we compared the efficacy of LPV/r and DRV/r combinations for the treatment of patients with COVID-19 during the second wave of pandemic. We found that the treatment with LPV/r was associated with better survival in hospitalised patients, but not with DRV/r. Comorbidities such as diabetes increased the risk of mortality in case of DRV/r but not with LPV/r. Lopinavir-ritonavir was particularly beneficial in patients with moderate-to-severe lesions on chest CT scan; however, arterial hypertension and the need for oxygen supplementation reduced its effectiveness.

Similarly to the HIV aspartic protease, the cysteine protease of SARS-CoV-2 was hypothesised to be a reasonable target of repurposed antiretroviral protease inhibitors (Magro et al., 2021). *In vitro* studies demonstrated the efficacy of lopinavir-ritonavir against SARS-CoV-2 and its ability to bind to SARS-CoV 3C-like protease, thus inhibiting viral replication (Zumla et al., 2016; Choy et al., 2020). Apart from lopinavir- ritonavir, another protease inhibitor combination darunavir-cobicistat was tested in silico and found to have theoretical affinity to 3C-like protease and potential inhibitory effect on viral replication (Sang et al., 2020). Later, an *in vitro* research demonstrated the lack of antiviral effects of darunavir on SARS-CoV-2 (De Meyer et al., 2020). In the early desperate times of the pandemic, protease inhibitors came into focus again after successful treatment of a patient with mild COVID-19 (Lim et al., 2020). Although lopinavir-ritonavir and hydroxychloroquine reduced organ support-free days and worsened the clinical outcomes among critically ill patients at an intensive care unit (Arabi et al., 2021), protease inhibitors remained still a potential option for hospitalised mild to severe COVID-19 patients. In a randomised, controlled, open-label trial by Cao et al., the authors suggested that lopinavir-ritonavir has no benefit in hospitalised patients compared to standard care (Cao et al., 2020a). Although the clinicians started to abandon lopinavir-ritonavir based on the results of this trial, several arguments should be taken into consideration: the trial was underpowered due to small sample size (N=199) and arguably, the treatment started at a median time of 13 days after symptom onset. Of note, the same trial reported several interesting findings with regard to the secondary outcomes suggesting lopinavir-ritonavir may be associated with reduced all-cause mortality (19% of patients in the lopinavir-ritonavir group vs. 25% of control group), decreased risk of severe adverse events (20% in lopinavir-ritonavir vs 32% of control group) and lower risk of severe respiratory failure (13% vs. 27%, respectively) (Dalerba et al., 2020). Another group reanalysed these data and found that lopinavir-ritonavir can contribute to clinical improvement (Carmona-Bayonas et al., 2020). In this aspect, our results were consistent with their observation that lopinavir-ritonavir reduced mortality. A further publication advised to consider starting lopinavir-ritonavir earlier and speculated about the favourable effects of LPV/r, supporting the need for further clinical studies in this field (Owa and Owa, 2020).

In the RECOVERY trial LPV/r was not associated with reduced 28-day mortality, duration of hospital stay or the risk of progression to invasive mechanical ventilation. However, we have to evaluate these results cautiously as an overall 74% of patients required respiratory support in form of oxygen therapy at baseline and both the usual care and LPV/r were started at an average 8 days after symptom onset (RECOVERY Collaborative Group, 2020). In line with this, oxygen therapy was associated with worse outcomes in the LPV/r group in our study. Additionally, the WHO SOLIDARITY trial included primarily patients already being ventilated or using respiratory support at the time of their recruitment.

Patients assigned to antiviral treatment had severe COVID-19, most of them lived in Asia and Africa where limited treatment resources were available, and some patients would have needed respiratory support (WHO Solidarity Trial Consortium, 2022). Although the final results concentrated on the effects of remdesivir therapy, the interim research results suggested that LPV/r can have benefits in a defined group of patients (< 50 years) (WHO Solidarity Trial Consortium et al., 2021). Further studies compared LPV/r to other drug combinations, and one of them found that a triple combination with LPV/r, interferon beta -1b and ribavirin significantly improved National Early Warning Score (NEWS2), Sequential Organ Failure Assessment (SOFA) scores, hospital stay and decreased the time to negative viral load in nasopharyngeal specimens only if the patient allocation was done prior to 7 days after symptom onset (Hung et al., 2020). These data also pointed out that similar to MERS and SARS, the early antiviral treatment may be crucial against SARS-CoV-2, and one should consider the wide randomisation windows in the evaluation of clinical trial results (de Wit et al., 2016). Additional studies aimed to compare LPV/r alone to other drugs such as umifenovir, novaferon or hydroxychloroquine, and to their combinations. However, these studies had no standard care group, had small sample size, poor statistical power and different control groups with various study population heterogeneity, therefore they have to be interpreted cautiously (Li et al., 2020; Nojomi et al., 2020; Zheng et al., 2020). The TOGETHER trial enrolled COVID-19 patients with at least one clinical criterion for high risk and compared the effects of LPV/r or hydroxychloroquine to placebo. Although the participants received the treatment less than 8 days from symptom onset, the trial confirmed that neither LPV/r, nor hydroxychloroquine showed associations with COVID-19 mortality or hospitalisation (Reis et al., 2021). Another study confirmed that all paediatric patients with mild or moderate COVID-19 receiving LPV/r were cured and had reduced hospital stay (Qiu et al., 2020). Taken together, these data suggest that LPV/r may have clinical benefits in a predefined subgroup of hospitalised patients without baseline respiratory support. Indeed, our data is consistent with this theory, as patients without severe comorbidities or oxygen supplementation receiving LPV/r showed association with lower risk of COVID-19 associated death.

Darunavir, another protease inhibitor, was identified as a promising hit by computational methods which indicated it to be more effective against COVID-19 than LPV/r (Khan et al., 2021). Darunavir was mostly used in combination with cobicistat (DRV/c), but this drug combination did not meet the initial expectations. DRV/c was effective at high concentrations against SARS-CoV-2 *in vitro* and proved to be more tolerable and safer than LPV/r (Orkin et al., 2013; Yamamoto et al., 2020). However, a study by Milic et al. confirmed that patients on DRV/c had higher mortality rate and risk for mechanical ventilation, compared to patients received standard care (Milic et al., 2021). A further study compared DRV/c with LPV/r and revealed that DRV/c was associated with 89% increased risk of death if the patients were women, older, had severe infection and received hydroxychloroquine. Additionally, a subgroup analysis of data from this study showed a lower risk of death in patients with mild disease treated with LPV/r (Di Castelnuovo et al., 2021). Another observational study comparing the efficacy of early administered DRV/c versus LPV/r unravelled that LPV/r was associated with faster time to recovery and virological clearance, but not DRV/c (Elmekaty et al., 2022). These findings can be also attributed to the unfavourable toxic side-effects of DRV/c (Hunt et al., 2011). The majority of darunavir is bound to plasma proteins and metabolised by CYP3A4. As ritonavir is a known CYP3A4 inhibitor, their combination can be theoretically a more efficacious drug against COVID-19 as DRV/c, however, there is an increasing risk of drug interactions and severe side-effects (Hsu et al., 1998; Rittweger and Arastéh, 2007). In fact, our study is the first that evaluated the clinical outcomes of DRV/r on COVID-19 patients, compared to LPV/r or standard care group. Based on our results, DRV/r was associated with increased mortality rates compared to LPV/r treated patients, showed higher rates of impaired glucose metabolism and oxygen support, but we did not observe higher frequency of cardiovascular alterations.

To our knowledge, our study is the first to compare the clinical effects of LPV/r and DRV/r protease inhibitors in hospitalised patients with mild-to-severe COVID-19, however, our results have some limitations. Firstly, we checked the effects in a retrospective observational nature. The treatment options were highly dependent on the accessibility of LPV/r and DRV/r in our centre. A placebo-controlled project would have been also not accepted during the early desperate time of the pandemic, so we tried to exclude the potential confounding factors with propensity-score matching method. Additionally, our study was launched relatively early during the pandemic and it would have been complicated to set up a randomised prospective study and get an ethical approval for these combinations.

## Conclusions

The main benefits of drug repurposing include the existing knowledge of a drug’s pharmacokinetics, pharmacodynamics, and toxicity. Employing similar strategies to find anti-SARS-CoV-2 compounds could significantly reduce the time required to identify an effective treatment for COVID-19, thereby lessening the disease’s impact, including hospital admissions, deaths, and long-term consequences. The global health crisis caused by the COVID-19 pandemic has necessitated the urgent acceleration of drug discovery and the swift identification of effective treatments and therapeutic options. While repurposed drugs must still undergo clinical trials, it is evident that this approach can quickly uncover effective treatments, even among those drugs that did not succeed for their initial intended use. Despite the widespread administration of COVID-19 vaccinations, COVID-19 continues to pose significant financial and public health challenges globally. Furthermore, vaccinations are not equally accessible to all populations; hence, repurposed drugs for the treatment of COVID-19 remain a viable option.

This retrospective study demonstrated that early treatment with lopinavir-ritonavir was associated with significantly improved survival and mortality rates compared to darunavir-ritonavir. Lopinavir-ritonavir exhibited a higher probability of survival compared to both the darunavir-ritonavir and standard care groups. Therefore, in the event of a future medication shortage during the COVID-19 pandemic, it would remain a favourable option for a carefully selected subgroup of patients. Despite some studies, the long-term effects of lopinavir-ritonavir in these patients await confirmation in a prospective, randomised, controlled trial. Our findings suggest that lopinavir-ritonavir should not be entirely disregarded for the treatment of COVID-19 and confirm that the combination of darunavir-ritonavir offers no additional clinical benefit for these patients.

## Data Availability

The raw data supporting the conclusions of this article will be made available by the authors, without undue reservation.
